# Intra-urban patterns of neighborhood-level social capital: a pilot study

**DOI:** 10.15171/hpp.2019.21

**Published:** 2019-05-25

**Authors:** Jaron King, Cassidy A. Hine, Tessa Washburn, Hunter Montgomery, Robert A. Chaney

**Affiliations:** ^1^Department of Public Health, Brigham Young University, Provo, USA; ^2^College of Fine Arts and Communications, Brigham Young University, Provo, USA

**Keywords:** Social environments, Urban spatial distributions, Neighborhood, Urban health, Social support

## Abstract

**Background: ** Social capital is a construct of interaction and social trust in one’s fellow community members. These interactions can provide a safety net for individuals in terms of information, social support, and adherence to social norms. While a number of studies have previously examined the relationship between social capital and health outcomes, few have examined the theparallel relationship of social capital and geographic "place" with respect to health outcomes.

**Methods: ** Considering social capital as facilitated by specific structures, we evaluate the relationship between neighborhood-level social capital and disability rates in a major Southern US city. Disability rates were collected through neighborhood-level data via the AmericanCommunity Survey (ACS) and compared to a geocoded map of neighborhood-level social capital measures during spring, 2016.

**Results: ** Higher social capital within a neighborhood coincided with lower disability rates in that neighborhood (r=-0.14, P=0.016) when compared to random assortment models.

**Conclusion:** Findings from this research add evidence to the value of the built environment, not only providing resources and shaping choices, but for facilitating important social relationships.

## Introduction

Social capital is a measure of “civic connections and social trust”^1^ or “the degree of interaction with and trust in one’s fellow citizens”.^2^ Social capital has been associated with improved outcomes in various sectors including economics, education, and public safety.^1-3^ It represents an important construct because individuals with higher social capital also have improved resilience after natural disaster, improved economic performance overall and easier transitions through life changes.^4-6^ It is therefore fitting for public health to be interested in social capital as a means of understanding and preventing disease. We piloted an exploration of how social capital varies across an urban landscape, as does a correlated health outcome.

Social capital is of particular interest to public health professionals because of its association with overall health.^7-9^ Research surrounding social capital has occasionally represented contradictory associations, such as in Birmingham, Alabama, when increased social capital corresponded with an increase in mental distress.^10^ However, increased social capital has also been associated with a variety of specific positive health outcomes including successful cocaine quit attempts,^11^ decreased binge drinking,^12^ lower obesity rates,^13^ and even better overall health for older individuals.^14^

Various theories have been proposed to explain the connection between social capital and health outcomes. Kawachi and Berkman proposed three possible explanations for the association between social capital and health outcomes.^15^ First, individuals share information through their social connections including health information. Those with higher social capital may receive more valuable health information. Second, social capital may also enforce healthy behavioral norms, such as physical activity and service-accessing behaviors. Finally, social capital may provide a support system that facilitates healthy living.

Each of these three proposed explanations focuses on individual-level interactions, which is consistent with the usual method for measuring social capital. Social capital is generally measured through individual surveys. Robert Putnam, one of the modern founders of social capital theory, directs the Social Capital Community Benchmark Survey, which measures 11 components of social capital through a lengthy questionnaire.^16^ In public health research, a survey created by Sampson et al is likely the most commonly used individual survey.^17,18^ Sampson et al measured social cohesion and informal social control through a 10-item questionnaire.^17^ A scale developed by Kawachi et al is also frequently used in public health research.^3,18^ Kawachi et al uses existing data from the General Social Surveys on group membership, perceived trust, and perceived norms of reciprocity, then aggregates this individual-level data to the state level.^3^

While Kawachi and Berkman^15^ focused on the individual-level influences of social capital, Hunter et al^19^ proposed an expanded explanation for the association between social capital and health outcomes. They suggest that social capital may mediate the broader effects of the social determinants of health. This proposal is supported by work from Kawachi et al which shows that social capital explains much of the differences in life expectancy, heart disease, infant mortality and self-reported health, even after adjusting for income.^3^

This view emphasizes the importance of social capital as a collective resource. Lochner et al similarly argued that social capital is a community characteristic and should be measured at the community level.^20^ They suggest direct observation of a community to measure social capital. However, the most effective way to measure social capital at the community level remains unknown. It is still debated, as how to quantify and measure social capital effectively given the context within each community is different and it may be difficult to apply the same variables across communities.^21^

Several studies have used the built environment as a possible way to measure social capital within communities.^22-25^ The idea that social capital is related to the built environment is founded upon this same environment providing context for social interactions—the relationship is reciprocal. Thus, measuring features in the built environment can act as a proxy for social capital since these are the places that facilitate social capital development. In this instance, geospatial analysis is an important tool to measure the spatial arrangement of the built environment and can help to identify potential spatial variation in social capital. This approach allows for the observation of individuals in the physical or social context in which they engage in daily activities that affect their health, i.e. considering settings.^26^

While social capital has been the object of growing interest in the past decades, no studies to this date have used geospatial mapping to examine the association between patterns of social capital and health outcomes. The purpose of this study was to determine the spatial patterns of social capital and its association with disability in the Atlanta, Georgia, USA metropolitan area. Disability rates remain an important public health metric as they represent both the success of public health in primary prevention of disability and the secondary focus of mitigating barriers to health for disabled persons.^27^

We sought to answer the following questions: (1) Is there a significant spatial pattern of social capital?; and (2) Is there spatial correlation between social capital and the health outcome disability? We hypothesize there would be a significant pattern of social capital based on Markeson and Deller’s^21^ research that demonstrates patterns of social constructs and the growing body of literature around spatial patterns of health behaviors, resources, and outcomes.

## Materials and Methods

### Data measures and acquisition

This cross-sectional study employed a purposive sampling method was used in Atlanta metropolitan area zip codes. Atlanta, is a diversified metropolis with a population of 420 003; a demographic profile of 40.1% white, 52.4% black or African American, 4.8% Latino, and 4% Asian; 24.0% of individuals were below poverty level.^28^ Community-level variables were identified from prior work by Markeson and Deller.^21^ The following community-level social capital variables were measured: places of worship, schools, physical fitness facilities, community centers, country clubs, labor unions, farmers markets, bowling centers, and political organizations. Some of the variables like “physical fitness facilities” had variant search terms, such as YMCA recreation centers, yoga studios, LA fitness, anytime fitness, recreation centers, etc.

Search terms for each variable and corresponding search terms entered into Google Maps. Physical structures that matched the specific variable criteria and fell within the circular metropolitan area of Atlanta (i.e. a 15-mile radius of downtown Atlanta) were included. Physical address and zip code were captured for each location. Each location was geocoded using the Google My Maps map creator to ensure it was within the 15-mile radius of downtown Atlanta. Data were collected during April, 2016.

Zip code-level census data were also collected, including demographics, transportation, health insurance coverage, and disability information gathered through the American Community Survey (ACS) for 2014 data.^29^ Zip codes that fell within the 15-mile boundary were included in the study. For the census data, zip codes were also excluded if they did not include any homes. These zip codes were primarily business or postal code, so no demographic data was available from the ACS. A full list of the variables and search terms used are found in Table 1.

### Data analysis

The address for each physical location variable were exported from Google My Maps and aggregated to zip codes using QGIS geographic information system.^32^ Social capital was reported as the density of social capital-promoting entities in the physical environment per zip code. A pattern of social capital across the Atlanta area was determined using Moran’s I test for global autocorrelation and local clusters of social capital were identified using Local Indicators of Spatial Autocorrelation (LISA).^33,34^ QGIS was used for spatial visualization and R statistical software was used for data analysis.^32,35^

## Results

We observed n = 287 zip codes in the Atlanta metropolitan region. The mean number of social capital “locations” across the region was 4.58 (Median = 2.0, min, Max = 0, 45). The mean social capital density was 1.50 locations/km^2^ (Median = 0.22 locations/km^2^, min, Max = 0, 75). The mean disability rate across this region was 9.20 (Median = 10.8, min, Max = 0, 18.6).

**Figure 1:**
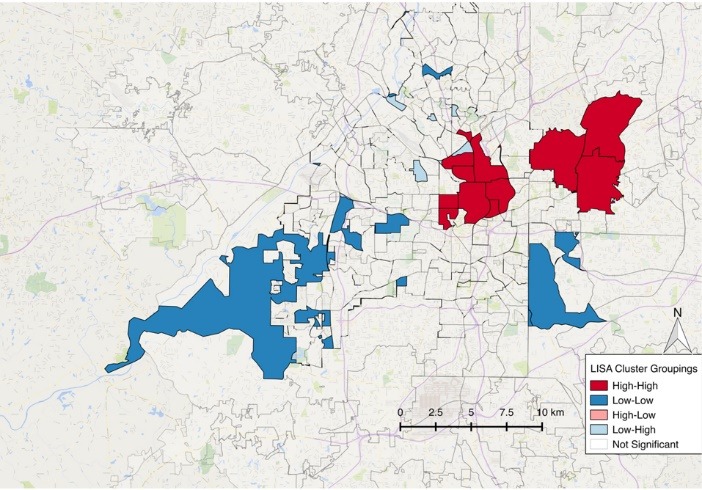
Social capital LISA analysis showing clustering pattern across the Atlanta region.

**Figure 2:**
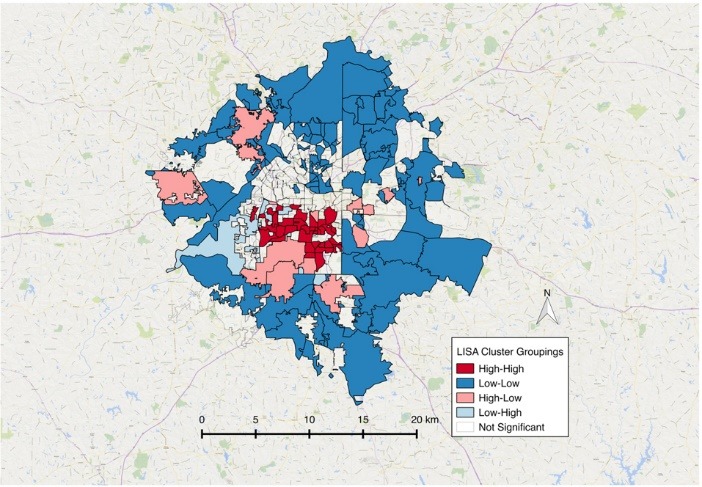
Disability LISA analysis showing clustering pattern across the Atlanta region.

**Figure 3:**
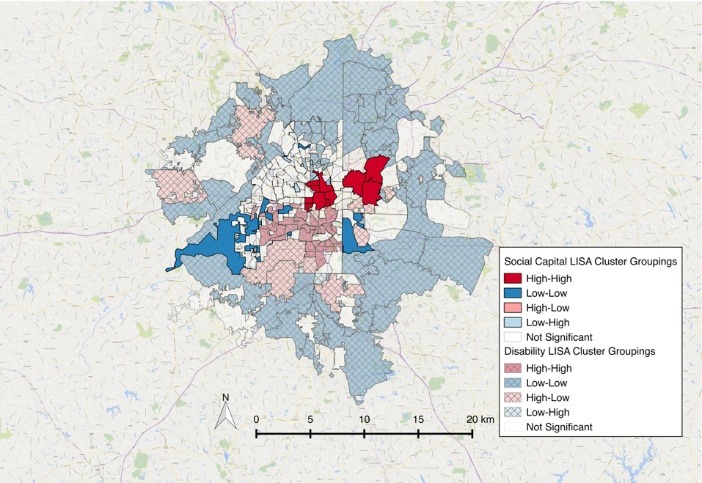
Overlay of social capital and disability clusters in Atlanta.

There was a significant global pattern for social capital clustering (I = 0.12, *P*<0.001) and disability (I = 0.31, *P* <0.001). Local clusters are presented in Figure 1 and Figure 2. The relationship between these two variables is presented in Figure 3 where there is a significant association between social capital variables and the percent of persons living with a disability in a discrete geographic location (e.g. neighborhood). Higher social capital within a neighborhood coincided with lower disability rates in that neighborhood (*r* = -0.14, *P* = 0.016) when compared to random assortment models.

## Discussion

We sought to determine if a significant spatial pattern of social capital existed in a large US city; and if such a spatial pattern existed, would it correlate with known patterns of health outcomes, in our case disability. As noted by Foster et al in 2015, there has been little research performed surrounding the question of spatial social capital variables.^36^ Our findings here further the utilization of spatial orientation within the social capital framework. GIS analysis of one major Southern U.S. city showed some correlation between spatial social capital variables and neighborhood-level (zip code) disability rates.

This study described *social capital* as a product the built environment, with physical locations serving as the primary metric of social capital. Regardless of the terminology used, we have established in this study that the greater number of physical structures enumerated here, the lower the disability rates in that location. Using physical locations as a measure of social capital we see that - whether through self-selection or some other process - individuals living with disabilities in this city live further from these locations than can reasonably be termed random. Thus, physical locations of social capital play some role in this specific health outcome. We suspect that observations from this study are indicative of other health and social capital relationships—that they vary across space and time, and are correlated with one another.

Many possibilities coalesce to create the connection between disability rates and social capital. For example, “shared knowledge” is an important asset in the framework of social capital, and these physical places allow people with physical and mental limitations to collaborate together to address both intra and interpersonal concerns. The formal disability rate may also be depressed by the ability of a community to provide necessary aid to individuals in need quicker than would be available through the State’s designation of “disabled.” Through these and other pathways locations of social capital combine to lower disability rates in this major city.

Social capital being a construct of civic connections and social trust, is also a protective factor that builds resilience. Resilience, being the ability of individuals or a community to use available resources to withstand risk factors, and social capital are then intertwined.^37^ The development of resilience is an important byproduct of social capital and has been document in other studies that report the value of social connections among transgender youth, non-medical use of prescription drugs, depression in the elderly, and others.^38-40^ As seen in these other scenarios, social connections provide opportunity to build resiliency, share knowledge, explore resources, and develop a “safety net” to rely on. That the resources to build social capital are not the same between neighborhoods can explain why social capital rates differ and are likely related to differing disability rates observed here.

This study’s limitations represent many of the same limitations common to social capital research. First, our study only examined one large US city at a single point in time. Likewise, it presents limited health outcomes data as this was a preliminary investigation. Future research should seek to compare multiple municipalities, as social capital is inherently tied to the built environment, the unique structures of each city are likely to produce unique social capital patterns. Additional examination of the spatial relationship between a wide variety of health outcomes and social capital will be insightful for understanding what conditions most benefit from heightened social capital.

As the 21st century push for “health in all policies” continues to gain traction, all aspects of community development should be influenced by the understanding that locations of social capital have a known benefit to the societies in which they are located.^41^ Increasing the density of social capital locations seems to benefit small and large neighborhoods alike. While specific measures of geographic social capital should be further investigated, the potential for health promotion to operate in both public and private space cannot be overstated.

## Ethical approval

No ethical considerations are declared.

## Competing interests

The authors declare that they have no competing interests.

## Funding

This study was unfunded.

## Authors’ contributions

JK, CH, TW, RC involved in conception and study design. TW performed data collection. CH and RC performed data analysis. HM provided background and acted as a corresponding author. All authors contributed to manuscript writing. JK and RC provided supervisory manuscript editing.

**Table 1:** Variables used to describe the presence of social capital by neighborhood

**Variables**	**Social Capital Rationale** ^21^
**Associational** - Bowling alleys - Civic and social associations - Physical fitness facilities - Public golf courses - Sports clubs, managers and promoters - Membership sports and recreation clubs - Political organizations - Professional organizations - Business associations - Labor organizations	Representing a location where (or vehicle for) community member interaction^30^
**Religious activity** - Places of worship - Percentage active in a religion	Representing locations where individuals can interact and build complex relationships^31^
**Cooperative organizations** - Number of arts cooperatives - Number of child-care cooperatives - Number of educational cooperatives - Number of grocery store cooperatives	Unites communities voluntarily - these four selected per specific focus on community needs
**Non-profits** - Number of non-profits	Non-profits are more likely to ensure a "service is available in the community regardless of the economic viability of the enterprise" (p. 62)
**Composite index** - Sum of associations above per 10 000 persons - Census mail response rate - Voter turnout (2008) - Total number of non-profits per 10 000 persons	“Goetz and Rupasingha used principal component analysis to combine several factors that could be associated with social capital into a scalar index” (p. 63)
